# Reversibly Desensitising the Human Retina to Delay Saccadic Reaction Time for Diagnostic Prototyping

**DOI:** 10.1007/s44402-026-00090-8

**Published:** 2026-05-07

**Authors:** Suzanna M. L. Janssens, Johan J. M. Pel, Peter Bremen

**Affiliations:** Department of Neuroscience, Erasmus MC, Rotterdam, The Netherlands

**Keywords:** Eye movement, Oculomotor behaviour, Perimetry, Photobleaching, Visual field

## Abstract

Glaucoma is a progressive retinal disease and a leading cause of blindness. Early detection is crucial but challenging due to subtle symptoms and the burden of standard clinical tests. Standard Automated Perimetry, though the gold standard for detecting glaucomatous functional deficits, is time-consuming and demanding for patients. Eye-movement perimetry (EMP) offers a promising alternative by using saccadic reaction times as indirect markers of visual-field integrity. However, EMP development is impeded by the need for large trial numbers due to inherent reaction-time variability and ethical constraints on patient involvement in early-stage prototyping. To address these limitations, this study employed photopigment bleaching to develop a method for generating reaction-time delays typically associated with glaucoma. Specific retinal regions in healthy participants were desensitised transiently using non-harmful light patterns displayed on a standard monitor. Participants performed an oculomotor task with targets presented at locations across the visual field either with or without desensitisation. Saccadic reaction times (target onset to movement onset) were measured to quantify the behavioural effect of desensitisation. Localised desensitisation significantly increased the mean (50–130 ms) and variability (20–60 ms) of saccadic reaction times in desensitised regions, and could lead to detection deficits resembling scotomas. These effects were more pronounced at greater eccentricities, consistent with retinal heterogeneity in photoreceptor density and receptive-field size, and decayed within hundreds of milliseconds (250–600 ms). This approach offers a safe, transient and spatially specific method for inducing reaction-time delays across the visual field in healthy individuals, enabling efficient prototyping of EMP paradigms prior to patient testing.

Key Points

- A reversible light-based method safely induces localised retinal desensitisation in healthy participants, effectively modelling the behavioural consequences of glaucoma, including increased reaction-time mean, increased variability and localised sensitivity loss.
- This technique enables rapid prototyping of reaction-time-based diagnostic tools, such as eye movement perimetry, while minimising the burden on clinical populations during early-stage test development.
- The approach offers a spatially precise model for studying the behavioural consequences of localised visual impairment relevant to early glaucoma detection.

## Introduction

Glaucoma is a progressive and irreversible neurodegenerative disease of the retina and a leading cause of blindness worldwide [1–3]. The disease primarily affects the optic nerve, leading to gradual vision loss, often without noticeable symptoms in its early stages [4]. As a result, many individuals remain undiagnosed until substantial visual-field defects emerge, significantly impacting quality of life [5, 6].

Visual-field testing is essential for detecting functional deficits associated with glaucoma. Standard automated perimetry (SAP) remains the clinical gold standard, yet the high demands it places on patient compliance and the lengthy nature of testing procedures are considered significant drawbacks. Consequently, alternative methods such as eye-movement perimetry (EMP) have been proposed to improve diagnostic efficiency and accessibility [7–13] (for non-latency-based EMP [14, 15]). EMP leverages reaction times measured from goal-directed saccades, which are slowed by 40–200 ms in patients with glaucoma. Saccadic reaction times, while being an indirect measure of visual-field integrity, potentially offer a faster and more user-friendly approach to glaucoma screening. EMP has shown promise in detecting glaucoma, but further refinement is necessary to enhance its accuracy and optimise its clinical utility.

One challenge for the clinical use of EMP is that reaction-time distributions are inherently right-skewed, complicating statistical analyses and necessitating large repetition counts for robust parameter estimation [16–18]. This requires long measurement sessions, particularly in early glaucoma. Moreover, the development and validation of fast and accurate reaction-time-based diagnostic paradigms are constrained by ethical and practical considerations. That is, it is undesirable to subject patients with visual impairments to prolonged experimental sessions during early-stage test development. A potential solution is to test new paradigms in a larger, more readily accessible participant pool, consisting of healthy individuals with normal vision. However, their saccades are usually faster and less variable than those of patients with glaucoma. We developed a method to reversibly increase saccadic reaction times and their variability in healthy participants by attempting to approximate reaction time properties typically observed in glaucoma. The intent with this method is to accelerate the development of reaction-time-based screening tools by reducing the burden and reliance on clinical populations.

The rationale behind the method is that decreased retinal sensitivity, evoked by desensitising the retina with bright light, can lead to slowed reaction times. This desensitisation results from a process called photobleaching, which is a transient light-induced reduction in the ability of retinal photopigments to absorb light [19]. Photobleaching has been a fundamental tool in vision science for nearly a century [19–23]. Classical studies provided a quantitative understanding of how light exposure affects visual sensitivity and recovery over time [24–26]. These experiments relied on strong light sources such as tungsten lamps, xenon arc lamps or controlled flashes from specialised equipment to induce full-field (Ganzfeld) retinal adaptation effects. Retinal recovery after photobleaching follows a logarithmic function, with the recovery rate depending on factors such as light intensity, duration of exposure and retinal area. Photobleaching studies on dark adaptation showed that cones regenerate more rapidly than rods [24] and that photopigment depletion decreases contrast sensitivity and increases visual detection threshold [21].

In contrast to the classical photobleaching studies that used strong light sources capable of depleting photopigments across a significant portion of the retina, we propose using much weaker light sources, such as thin-film transistor (TFT) screens found in standard computer monitors. This approach offers several advantages. First, unlike classical xenon or tungsten lamps (luminance levels of 10^6^–10^8^ cd/m^2^), TFT screens emit significantly weaker light levels (peak luminance levels of ~250–400 cd/m^2^), thereby reducing the risk of retinal damage or long recovery periods. Second, while classical methods desensitise a large retinal area, TFT screens allow for controlled localisation of retinal desensitisation by displaying bright light in customisable spatial patterns. For example, when using a screen-based presentation of a white shape against a black background, the size and position of the shape can influence which specific region of the retina is desensitised. Third, TFT-based desensitisation protocols can be easily implemented in standard laboratory or clinical settings, requiring only software adjustments rather than specialised high-intensity light sources. Importantly, the limited peak luminance of TFT screens provides a safe operating range without permanently affecting photoreceptor function.

 This study demonstrates that it is possible to reversibly increase saccadic reaction times and their variability in selected visual-field regions of participants with normal vision by exposing them to bright, two-dimensional patterns of non-harmful light for a short time.

## Methods

### Participants

Six participants aged between 24 and 45 years (median: 28 years; 2 females) were included. None of the participants had ever been clinically diagnosed with an ophthalmic condition or had reported any symptoms indicative of uncorrected refractive error or ocular pathology. An exception was participant 2, who had a known refractive error but did not wear their prescription glasses during the experimental sessions (right eye: –3.25 DS/–0.75 DC × 180; left eye: –3.00 DS/–1.75 DC × 175). Participants 1 and 2 were authors of this study.

### Setup

All measurements were conducted in a dark and quiet room. Stimuli were presented on a 27-inch Iiyama Prolite XB2776QS monitor (resolution: 2560 × 1440 pixels, refresh rate: 60 Hz; contrast and brightness set to 100%; iiyama.com) controlled via an HP Z2 G5 Mini i7 (hp.com) computer running custom-written MATLAB software (Version R2023b MathWorks, 2023; mathworks.com) and using the Psychtoolbox (Version 3.0.19; [27–29] psychtoolbox.org) for stimulus generation.

The position of both eyes was sampled at a frequency of 60 Hz with infra-red-corneal-reflection eye tracking via a Tobii Pro Nano eye tracker (Version 2.5; Tobii AB, 2023; tobii.com) mounted on the monitor. The Tobii Pro Software Development Kit for Windows (SDK version 1.11.0.1) was used to control the acquisition of gaze data in MATLAB. A forehead-and-chin rest was used to stabilise the participant’s eye position at 60 cm from the screen’s centre.

### Visual Stimuli Common to All Experiments

For all experiments, three types of visual stimuli were presented against a black background with a luminance of 6 cd/m^2^: an achromatic fixation circle (*r* = 0.25°, 350 cd/m^2^, Weber Contrast = 57), a target circle with varying luminance values (*r* = 0.25°; see details for Experiments 1 and 2 below) and an achromatic, ring-shaped area (350 cd/m^2^, Weber Contrast = 57) centred at the fixation circle for desensitising the retina at different eccentricities (see below). Luminance values of the fixation circle, target circle and desensitising area were measured at eye level using a lux meter (93560 Luxmeter; BEHA-Amprobe GmbH; beha-amprobe.com) at 60 cm and were derived from the instrument’s ‘cd mode’ setting.

All locations were defined using a polar coordinate system with its origin at the centre of the screen. The participant’s cyclopean eye, the point located between the two eyes, was horizontally and vertically aligned with the screen’s centre at a distance of 60 cm. Eccentricity (*R*) was defined as the radial distance from central fixation, measured in visual angle subtended from the participant’s cyclopean eye. Direction (*ϕ*) was measured relative to the horizontal meridian, with 0°and 180° indicating locations to the right and left of the centre, respectively.

### Paradigm and General Procedure

**Fig. 1:**
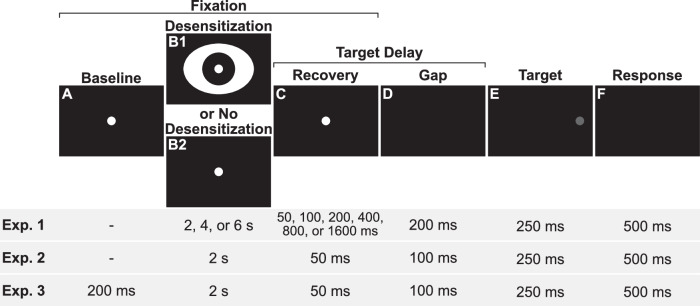
General trial structure and temporal sequence of the visuomotor paradigm used in all three experiments. *Top*: Sequence of events. During the fixation period (**A**, **B1**, **B2**, **C**), participants maintained fixation on the white fixation circle. Only experiment 3 started with a baseline fixation period of 200 ms (**A**). Retinal sensitivity of a circumscribed area was manipulated by either presenting (**B1**) or not presenting (**B2**) a desensitisation area for a variable duration. Shown here is is a schematic of the area used for experiments 1 and 2. See Fig. 2 for the areas used in experiment 3. A recovery period followed, during which the fixation circle remained visible for a variable duration (**C**). Next, the fixation circle disappeared and after a temporal gap (**D**) the target was presented (**E**). Target delay was determined by the durations of the recovery (**C**) and gap (**D**) periods. The response period ended after an interval of 500 ms (**F**). *Bottom*: Table summarising the duration parameters for all periods and experiments. Exp. = experiment.

Figure 1 illustrates a generic trial sequence for all experiments. Each trial started with an achromatic fixation circle (Fig. 1A and B), either with (Fig. 1B1) or without (Fig. 1B2) a desensitisation area. Fixation duration (Fig. 1A–C) varied depending on the experiment. To elicit fast responses, a short gap followed the disappearance of the fixation circle (Fig. 1D). Subsequently, the target circle appeared for 250 ms (Fig. 1E). After a response delay of 500 ms (Fig. 1F), the next trial began. Trials with and without desensitisation and all experimental parameters (fixation and desensitisation durations, stimulus locations and luminance) were pseudo-randomly interleaved and each parameter setting was presented with and without desensitisation.

For all experiments, participants were instructed to fixate upon the fixation circle until a target appeared. Upon target perception, participants had to make a saccade towards the perceived target location and re-fixate the fixation circle when it appeared again. Consistent with standard speed–accuracy trade-off paradigms, the instructions were to look at the targets as quickly and accurately as possible [30]. Participants completed the experiments over multiple days in sessions of ~50 min. In one session, participants completed about eight blocks (see below), each lasting around 5 min, with short breaks allowed between blocks. Before each block, the eye tracker was calibrated with a nine-point-grid calibration routine to convert eye position in normalised screen coordinates to degrees.

### Experiments

#### Experiment 1—Desensitisation Time Course

Experiment 1 determined the time course of recovery from retinal desensitisation by varying the duration of the desensitisation period (*t*_des_ = {2, 4, 6} s; Fig. 1B) and target delay (*t*_delay_ = {0.25, 0.3, 0.4, 0.6, 1, 1.8} s; Fig. 1C and D). The fixation circle was located at (*R*, *ϕ*) = (0, 0)° and targets (6.6 cd/m^2^) appeared at *R* = {5, 10, 20}° and *ϕ* = {0, 180}°, resulting in six possible target locations. The desensitisation area was an elliptical annulus centred on the fixation circle at (*R*, *ϕ*) = (0, 0)°. The elliptical annulus was generated by a white desensitising ellipse (major axis: 15°, minor axis: 10°) with a central black masking circle (radius *r* = 6°) overlaid to protect the central visual field. This configuration created a desensitised area spanning from an inner radius of 6° to an outer elliptical boundary of 15° × 10°. Consequently, only targets at *R* = 10° were presented within the desensitisation area. In contrast, the targets at 5° fell within the central black mask and targets at 20° were located entirely outside the boundaries of the desensitising area, allowing both to serve as internal controls. Visual schematics of this spatial arrangement and the resulting target overlaps are provided in the pictograms of Figs. 1B1, 4 and 5. The gap between fixation and target onset was 200 ms. Experiment 1 comprised 2160 trials (three desensitisation periods × six target delays × six target locations × two desensitisation conditions (present or absent) × 10 repetitions), divided into 40 blocks. Participants completed the experiment in five sessions, with trials from each repetition grouped together in four successive blocks.

#### Experiment 2—Target Luminance

Experiment 2 characterised the interaction of desensitisation and target luminance on reaction times. Only participants 1 and 2 took part in this experiment. The trial structure, target location and desensitisation area were identical to those used in Experiment 1. However, the desensitisation and recovery periods were fixed to 2 s (Fig. 1B1/2) and 50 ms (Fig. 1C), respectively, and the gap (Fig. 1D) was set to 100 ms to minimise search behaviour. Targets were presented at a luminance of *L* = {6.3, 6.6, 7.3, 8.8, 10.8, 13.2} cd/m^2^ (Weber contrasts = {0.05, 0.10, 0.22, 0.47, 0.80, 1.20}). This resulted in a total of 1440 trials (six target luminance values × six target locations × two desensitisation conditions (present or absent) × 20 repetitions), which were divided into 20 blocks. Participants completed the experiment in one session, with each block containing one repetition of all conditions.

#### Experiment 3 –Spatial Mapping

**Fig. 2:**
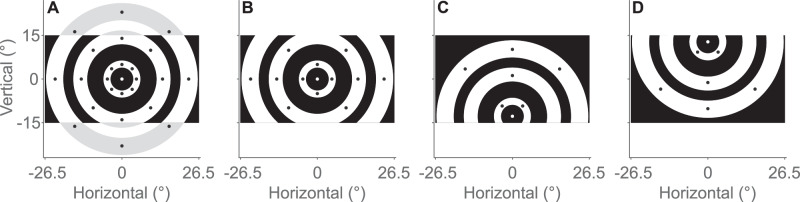
Schematic overview of spatial configurations in Experiment 3. Black rectangles depict the background corresponding to the active display area of the TFT monitor. White markers indicate the fixation location in all panels. Black markers represent all possible target locations. White concentric rings at eccentricities of 5°, 14° and 23° illustrate the desensitisation areas (centred on fixation). **A** All 24 possible target locations in oculocentric coordinates (*R* = 5°, 14°, 23°; *ϕ* = 0°, 45°, 90°, 135°, 180°, 225°). **B–D** Target locations in screen-centred coordinates with central fixation (**B**: 0°, 0°) and eccentric fixation positions (**C**: 0°, −13° and **D**: 0°, 13°). Due to monitor size constraints, eccentric fixation positions were required to present targets at larger retinal eccentricities while keeping them within display boundaries. The number of available target locations differed between central (12 targets; **B**) and eccentric fixations (six targets; **C** and **D**). Note that this figure is schematic. In the experiment, only one target and, if applicable, only one desensitisation area were presented per trial. The figure summarises all possible spatial configurations.

Experiment 3 characterised the desensitisation effect on reaction times in two spatial dimensions across multiple eccentricities. The trial structure followed Experiment 2, with the addition of a 200 ms baseline period at the beginning of the trial (Fig. 1A). Targets (6.6 cd/m^2^; Weber contrast: 0.10) were presented at 24 locations across the visual field at *R* = {5, 14, 23}° and *ϕ* = {0, 45, 90, 135, 180, 225}° (Fig. 2A; black markers).

Due to the limited size of the TFT monitor (black area in Fig. 2), systematic adjustment of the fixation position was necessary to present targets at more eccentric retinal locations (Fig. 2B–D). Accordingly, the fixation circle was not only presented at (*R*, *ϕ*) = (0, 0)° (Fig. 2B), but also at (*R*, *ϕ*) = (0, –13)° (Fig. 2C) and (*R*, *ϕ*) = (0, 13)° (Fig. 2D). Differences in target distributions for central (12 targets; Fig. 2B) and eccentric (six targets; Fig. 2C, D) fixations can impact saccadic parameters such as reaction time [31, 32]. These effects were equally present in trials with and without desensitisation, and did not impact direct comparisons between these conditions.

Desensitisation areas were presented at each target eccentricity (*R* = {5, 14, 23}°). They were created by overlaying a black circle (*R* = {4, 12, 20}°) on a white circle (*R* = {6, 17, 26}°), both centred around the fixation location. Experiment 3 comprised 1920 trials (24 locations × four desensitisation conditions × 20 repetitions) distributed across 20 blocks, so that each block contained one repetition of all conditions. Participants completed the experiment in four sessions of five blocks.

### Data Analysis

#### Saccade Analysis

In a minority of trials, eye tracking was disrupted resulting in signal loss and discarded trials (technical exclusion: Exp. 1: <1%, Exp. 2: 6%, Exp. 3: 5%). For all other trials, a custom-written MATLAB programme was used to post-process the calibrated eye-tracker traces. Traces from the left and right eye were averaged and up-sampled to 1 kHz using a modified Akima algorithm (‘makima’ method for MATLAB’s ‘interp1’ function) [33, 34]. This algorithm provides smooth, continuous interpolation without introducing oscillations or overshooting, preserving the stereotypical shape of saccadic traces. Eye blinks were identified by firstly calculating the two-dimensional velocity profile of the trace as the Euclidean norm of the horizontal and vertical eye position derivatives, divided by the time interval between consecutive samples. Secondly, using a velocity criterion of 600°/s, blink events were identified and smoothed with a three-sample running average before interpolating (‘makima’ method) between the onset and offset of the event.

From the post-processed traces, saccades and their parameters (onset, offset, starting and endpoint locations and reaction time) were extracted as follows. Peaks (‘findpeaks’ function with a minimum peak distance of 200 ms, i.e., required temporal separation between successive local maxima on the Euclidean velocity profile) were detected on the velocity trace with a two-step velocity criterion to (1) identify a candidate saccadic event (≥100°/s) and (2) to localise the onset and offset of the saccade (≥50°/s). Saccade selection was visually inspected and corrected if necessary. Reaction time was defined as the time difference between target and saccade onset times. For reaction-time analysis, saccades were included that met the following criteria: (1) first saccade after stimulus onset in a trial, (2) reaction time longer than 150 ms to exclude anticipatory responses, (3) Euclidian distance between the locations of the fixation circle and the starting location of the eye within three times the interquartile range [35] of the Euclidean distance distribution, calculated in a window of ±10 ms around fixation circle offset across all trials per participant, (4) goal-directed saccade as assessed by clustering the endpoints and (5) saccades with reaction times ≤600 ms based on visual inspection of reciprobit plots (<0.1% of trials across all experiments).

For the participant-wise selection of goal-directed saccades (criterion 4), the endpoint clusters for the trials without desensitisation were first determined by applying *k*-means clustering (MATLAB, ‘kmeans’) to the two-dimensional saccade endpoint coordinates, using the known target locations as initial cluster centres (*k* = number of targets). Endpoints assigned to the cluster seeded at their instructed target location were classified as goal-directed saccades, whereas all others were discarded. The resulting cluster centroids were then used as centres for clustering endpoints in trials with desensitisation. This approach avoids imposing an explicit spatial tolerance threshold and accounts for saccadic undershoot and participant-specific idiosyncrasies.

Selection criteria (3) and (4) were reviewed and corrected if necessary. Applying the above behavioural criteria excluded 5%, 2.5% and 22% of trials from Experiments 1–3, respectively. In Experiment 3, 92% of excluded trials came from trials with desensitisation, for which a large portion (59%) was not goal-directed.

#### Reaction-Time Analysis—Obtaining Sample Mean and Standard Deviation

Reaction-time distributions are typically skewed rightwards because fast responses occur more frequently than slow responses [17, 31, 36]. To meet the assumptions required for parametric comparisons, the data were normalised by transforming saccadic reaction times to promptness (*P* = 1/*RT*). The normality of the promptness distribution was assessed using a reciprobit plot [13, 36], and a linear regression line (‘regress’ function in MATLAB) was fitted to extract the sample mean (50% probability point) and standard deviation (slope). For ease of interpretation, the obtained promptness parameters (*μ*_P_ and *σ*_P_) were transformed back to reaction time by calculating the reciprocal of promptess, 1/*μ*_P_. However, due to Jensen’s inequality [37], 1/*μ*_P_ is generally smaller than the true reaction time mean, *μ*_RT_:

1 $${\mu }_{{{\rm{RT}}}}\ge \frac{1}{{\mu }_{{{\rm{P}}}}}$$

Similarly, the promptness standard deviation (*σ*_P_) was transformed to reaction-time standard deviation (*σ*_RT_) by

2 $${\sigma }_{{{\rm{RT}}}}\approx \frac{{\sigma }_{{{\rm{P}}}}}{{\mu }_{{{\rm{P}}}}^{2}}$$

The error introduced by these approximations is negligible if the promptness distribution is approximately normal with low variance, which was the case for the current data.

#### Reaction Time Analyses—Bootstrap Estimation and Permutation Testing

Estimation statistics [38, 39] was used to calculate the differences in promptness parameters (mean and standard deviation) between conditions with and without desensitisation.

To quantify the group-level effect of the desensitisation condition (D), relative to the no-desensitisation condition (ND), a hierarchical bootstrap procedure was employed that preserved both trial-level variability and participant-level structure. Within each of 1000 bootstrap iterations, trials were resampled with replacement separately for each participant and condition, and a bootstrapped mean reaction time was computed for conditions ND and D. For each participant, the condition difference (D−ND) was calculated from these bootstrapped means. These within-subject differences (one per participant) were then averaged to obtain a group-level effect size for that iteration. Repeating this procedure 1000 times yielded a bootstrap distribution of the group-level mean difference from which the point estimate is reported, as well as the 95% confidence intervals (CIs) and the margin of error (MoE), as the average length of the two CI arms.

To assess statistical significance for Experiments 1 and 3, a permutation *t*-test was performed on the original (non-bootstrapped) data. Specifically, the mean difference in D−ND was computed per participant, and the observed group-level mean was compared to a null distribution generated by randomly flipping the sign of individual participant differences across 10,000 permutations. Because the smaller participant pool in Experiment 2 (*n* = 2) provided an insufficient number of permutations to generate a valid null distribution, Welch’s *t*-test was employed for comparing distribution means and Levene’s test for comparing variances. These parametric tests were specifically selected for their robustness in handling high trial counts (1440 per participant) and unequal variances, a critical requirement given the significant increase in reaction-time variability induced by retinal desensitisation.

To improve interpretability, all results are reported as reaction times in ms instead of promptness values (see Eqs. (1) and (2)). Estimation statistics are reported as “difference ([lower CI bound, upper CI bound], MoE, *p*-value)”. A positive difference between D and ND indicates that reaction time increased with desensitisation. If the CI of the difference does not overlap with 0, then the compared distributions are considered significantly different from each other. The statistical analyses were conducted using a significance level (alpha) set to 0.05.

#### Statistical Analysis—Linear Mixed-Effects Models

To assess the impact of retinal desensitisation on visual responsiveness, linear mixed-effects models (LMEMs) were employed for both promptness (1/RT) and detection rate. All models were fitted using maximum likelihood (ML) estimation. To maintain a full-rank model, baseline ND trials were assigned the constant reference levels of the temporal factors (e.g., 2-s duration and 250-ms delay), allowing the model to estimate desensitisation effects as additive deviations from this unified baseline.

To account for nested data structures and individual differences, random intercepts were included for Participant, experimental Block and Target Direction (1∣Participant, 1∣Block, 1∣TargetDirection). The specific fixed effects for each experiment were as follows. For Experiment 1 fixed effects were Desensitisation (D vs. ND), Target Eccentricity, Desensitisation Duration and Target Delay, with a Desensitisation × Eccentricity interaction. For Experiment 2 fixed effects were Desensitisation, Target Eccentricity and Target Luminance, with a Desensitisation × Luminance interaction. And for Experiment 3, for both promptness and detection rate, predictors included Desensitisation Eccentricity and Target Eccentricity and their interaction. The detection rate model omitted the Block random intercept, as rates were calculated across all repetitions.

Corner-point (dummy) coding was employed for all categorical predictors to define a unified baseline for the estimates. Under this convention, the model intercept represents the predicted promptness when all predictors are at their reference levels. For instance, in the case of experiment 1, this would be a trial with no desensitisation at 10° eccentricity. To maintain a mathematically stable, full-rank model, trials in the ‘no-desensitisation’ condition were assigned the constant reference levels of the temporal factors (e.g., 2-s duration and 250-ms delay) as a baseline. This ensures the intercept serves as a ‘ground truth’ for normal performance, allowing the model to calculate the initial desensitisation effect and any subsequent variations in duration or delay as additive deviations from this single starting point. The significance of all fixed effects and interactions was determined using *F*-tests and *t*-tests with a significance level (*α*) set to 0.01 to minimise Type I errors.

The following Model Validation summary is provided for the sake of methodological completeness and narrative efficiency. Across all three experiments, the random effects for participant, experimental block and target direction accounted for a marginal portion of the total variance. Standard deviations for these intercepts remained consistently low, typically ranging from 1 to 9 ms (or ~9% for detection rates), indicating that while subtle individual differences and session-related fluctuations existed, they did not dominate the experimental effects. In contrast, the substantially higher residual standard deviations (e.g., 23–26 ms) confirm that the majority of variability in promptness was attributable to trial-level stochastic noise. This minimal influence of random factors justified the pooling of data across symmetric target directions for the primary analyses.

## Results

### Experiment 1—Desensitisation Time Course

#### Individual and Group-Level Reciprobit Analysis

**Fig. 3:**
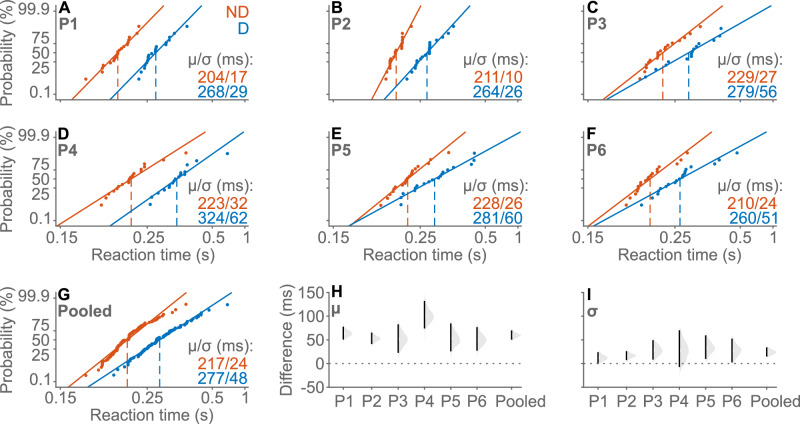
Effects of 2-s light desensitisation on reaction times to targets presented within the desensitisation area at 10° eccentricity after a 250 ms target delay. **A**–**F** Reciprobit plots (cumulative probability vs. promptness) for individual participants (P1–P6). Note that the abscissa is linear in promptness (s^−1^) but labelled with reaction- time values for easier interpretation. Reaction times from trials without desensitisation are shown in red (ND), whereas trials with desensitisation are shown in blue (D). Solid lines represent linear fits to promptness distributions and dashed lines indicate mean reaction time. Inset text indicates the reaction time mean (*μ*) and standard deviation (*σ*) in ms. **G** Reciprobit plot for data pooled across all participants. **H** and **I** Estimation statistics mean-difference plots for reaction time mean and standard deviation (difference: desensitisation–no desensitisation). Patches depict the bootstrapped difference distributions with white notches and black lines showing the mean and 95% confidence interval, respectively.

Figure 3 provides an overview of the effect of light desensitisation on reaction times for targets presented at 10° eccentricity (within the desensitisation area) with an initial 2-s period either without (red) or with (blue) desensitisation, followed by a 250-ms fixation period before target onset. The data are presented in reciprobit plots per participant (Fig. 3A–F) with the abscissa representing promptness labelled as reaction times to improve readability [36]. For all participants, desensitisation increased both the mean and standard deviation of reaction time (Fig. 3), in line with expectations. The smallest and largest increases in mean were 50 ms (P3 and P6) and 101 ms (P4), respectively. The smallest and largest increases in standard deviation were 12 ms (P1) and 34 ms (P5), respectively. Estimation statistics were used to assess whether these increases were significant. Figure 3H and I show the resulting mean-difference plots. A positive value indicates a reaction-time increase in the desensitisation condition. For all participants, increases in mean were statistically significant, with none of the 95% confidence intervals overlapping with 0 ms. Increases in standard deviation were statistically significant for all participants except for P4 (28 ms, 95% CI = [–8, 70] ms).

To provide an overview of group-level effects, responses were pooled across all participants and the corresponding reciprobit plots constructed for conditions with and without desensitisation (Fig. 3G). Note that the pooled distributions are normally distributed, as evidenced by their closely following the unity line. On a group level, desensitisation increased mean and standard deviation significantly as evidenced by the estimation statistics (Fig. 3H and I, Pooled), which showed a difference in reaction time mean and standard deviation of 62 ms (95% CI = [52,72] ms, MoE = 10 ms, *p* = 0.03) and 24 ms (95% CI = [15,34] ms, MoE = 10 ms, *p* = 0.03), respectively. This indicates that light desensitisation increased reaction time and reaction-time variability, both at the individual and group level. Note that for Experiments 2 and 3, individual reciprobit and mean-difference plots were reviewed as described here before responses were pooled across participants.

#### Temporal Dynamics of Recovery from Light Desensitisation

**Fig. 4:**
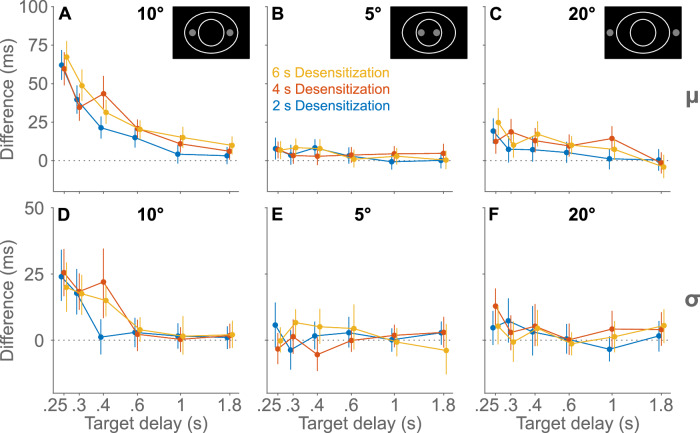
Effect of desensitisation duration on differences in saccadic reaction time (RT) across target delays. Differences (RT_desensitisation_–RT_no desensitisation_) are shown as a function of target delay for targets presented at 10° (**A, D**), 5° (**B, E**) and 20° (**C, F**) eccentricity. **A–C** Differences in means (‘*µ*’). **D**–**F** Differences in standard deviations (‘*σ*’). Pictograms show schematics of desensitisation area (white outlines) and target locations (grey circles). Data for three desensitisation durations, 2 s (blue), 4 s (red) and 6 s (yellow), are presented. Markers represent the difference and bars indicate the error-distribution 95% confidence intervals. Positive values reflect slower responses in the desensitisation condition. Data points have a slight horizontal offset to improve readability.

The effect of desensitisation duration, target eccentricity and target delay on reaction times were assessed with estimation statistics. Figure 4 depicts the pooled difference (marker) between desensitisation and no desensitisation and the 95% confidence intervals (error bar) for reaction time mean and standard deviation for all 54 conditions (three desensitisation periods × six target delays × three target eccentricities).

With a target delay of 250 ms, reaction times increased by ~60 ms for all desensitisation durations when targets were presented in the desensitisation area (10° eccentricity; Fig. 4A), in line with the pooled mean-difference plot presented in Fig. 3H. With increases in target delay, the difference between desensitisation and no desensitisation decreased systematically. For a desensitisation duration of 2 s, the difference was no longer significant after 1 s (4 ms, 95% CI = [–2, 10] ms, MoE = 6 ms, *p* = 0.06), indicating fast retinal recovery from desensitisation. The difference, although small, remained significant up to the largest target delay for both the 4-s (11 ms, 95% CI = [5,16] ms, MoE = 6 ms, *p* = 0.03) and 6-s (15 ms, 95% CI = [8,22] ms, MoE = 6 ms, *p* = 0.03) desensitisation durations, indicating a longer time constant for retinal recovery. Reaction time variability (*σ*) recovered faster than the mean (Fig. 4D). Variability remained increased for up to 300 ms (18 ms, 95% CI = [10,27] ms, MoE = 10 ms, *p* = 0.03) after 2 s of desensitisation and for up to 400 ms with desensitisation durations of 4 s (22 ms, 95% CI = [8,35] ms, MoE = 13 ms, *p* = 0.03) and 6 s (15 ms, 95% CI = [9,21] ms, MoE = 6 ms, *p* = 0.03).

Targets presented outside the desensitisation area (5° and 20° eccentricity) showed minimal saccadic reaction-time increases. Centrally located targets at 5° exhibited a maximum mean increase of only 8 ms, while peripheral targets at 20° showed larger but still limited spill-over effects (up to 24 ms) primarily after longer exposures and shorter target delays. Notably, saccadic reaction-time variability did not increase in any non-collocated condition (lowest *p* = 0.06 for targets at 20° after 6 s desensitisation and a 250-ms target delay). These findings confirm that the primary saccadic reaction-time effect is spatially specific to the desensitised retinal area.

To assess the effects of desensitisation and the temporal dynamics of recovery, saccadic reaction times were analysed using a linear mixed-effects model (Table 1). Under baseline conditions, representing targets at 10° eccentricity without desensitisation, the predicted reaction time was ~222 ms. Oculomotor performance was influenced by target eccentricity, as reaction times were negligibly slower (~2 ms) at 5° but significantly slower (~27 ms) at 20° eccentricity compared with the 10° baseline.

**Table 1:** Summary of fixed effects and the interaction effect from the linear mixed-effects model for Experiment 1 predicting promptness (1/s).

Fixed effects	*β* (1/s, ms)	SE	95% CI (1/s)	*p*
(Intercept)	4.51, 222	0.058	[4.40, 4.62]	<0.001
Desensitisation	–0.50, 27	0.015	[–0.53, –0.47]	<0.001
Target eccentricity (ref = 10°)				
5°	–0.04, 2	0.015	[–0.06, –0.01]	0.02
20°	–0.48, 27	0.015	[–0.51, –0.45]	<0.001
Desensitisation × Target eccentricity (ref = No desensitisation × 10°)				
Desensitisation × 5°	0.41, –19	0.021	[0.37, 0.45]	<0.001
Desensitisation × 20°	0.34, –16	0.021	[0.30, 0.38]	<0.001
Desensitisation duration (ref = 2 s)				
4 s	0.03, –2	0.011	[0.01, 0.05]	0.002
6 s	0.04, –2	0.011	[0.02, 0.06]	0.001
Target delay (ref = 250 ms)				
300 ms (smallest difference)	0.06, –3	0.015	[0.03, 0.09]	<0.001
1800 ms (largest difference)	0.23, –11	0.015	[0.20, 0.26]	<0.001

The intercept represents the baseline performance at the reference levels (no desensitisation, targets at 10° eccentricity, 2 s no-desensitisation period and a 250 ms target delay), with all other coefficients representing additive deviations from this state. Significant effects (*α* = 0.01) highlight the spatial and temporal parameters that influenced saccadic reaction times most robustly.

Retinal desensitisation significantly impaired performance, increasing reaction times by an average of ~27 ms. This effect exhibited high spatial specificity. The interaction term showed that reaction time in the presence of desensitisation was significantly faster at both 5° (~19 ms) and 20° (~16 ms), compared with the 10° reference level.

The duration of the desensitising light exposure (4 or 6 s) had a negligible effect on performance, resulting in functionally irrelevant changes (~2 ms) relative to the 2-s baseline. In contrast, the timing of the target onset revealed a robust recovery process. Saccadic responses became progressively faster as the target delay increased, showing a modest recovery of ~3 ms at the 300-ms delay and a more substantial recovery of ~11 ms by the 1800-ms delay.

These results demonstrate that retinal desensitisation induces a quantifiable and spatially specific delay in oculomotor responses that recovers rapidly over time. Based on these findings, a 2-s desensitisation duration and a 250-ms target delay were identified as the optimal parameters for subsequent experiments, as they provided a robust effect size while maintaining efficient experimental throughput.

#### Experiment 2—Desensitisation Effects Depend on Target Luminance and Eccentricity

**Fig. 5:**
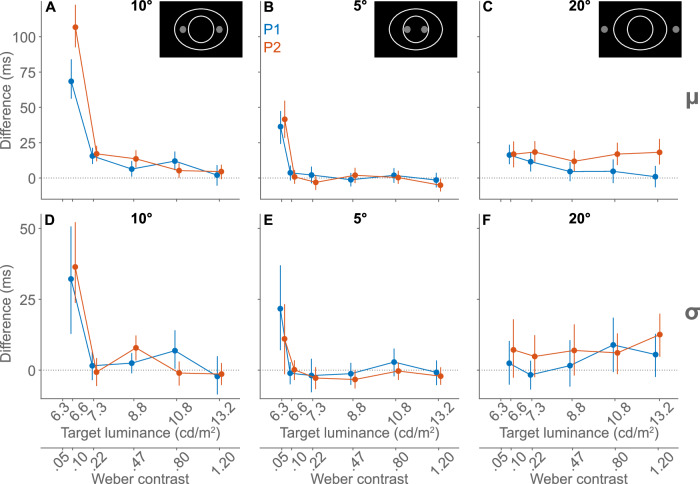
Effect of desensitisation on differences in reaction time across target luminance (abscissa in logarithmic scale). Differences (RT_desensitisation_–RT_no desensitisation_) are shown for targets presented at 10° (**A** and **D**), 5° (**B** and **E**) and 20° (**C** and **F**) eccentricity. Differences in means (‘*µ*’) and standard deviations (‘*σ*’) are depicted. Pictograms show schematics of desensitisation area (white outlines) and target locations (grey circles). Data are shown for two participants (P1 = blue, P2 = red). Desensitisation duration was 2 s and target delay was 150 ms. Markers represent the differences and bars the error-distribution 95% confidence intervals. Positive values reflect slower responses following desensitisation. Data at the lowest target luminance (6.3 cd/m²; Weber contrast = 0.05) are only depicted in panels **B** and **E**. In all other conditions at this luminance, desensitisation induced a functional scotoma so strong that participants did not make enough goal-directed saccades for the calculation of reaction-time differences.  RT reaction time.

Figure 5 shows the difference in reaction-time mean (top row) and standard deviation (bottom row) between trials with and without desensitisation across target luminance levels. Each panel displays the responses of participant 1 (blue) and 2 (red), with markers indicating the differences and bars the 95% confidence intervals of the error-distribution. At the lowest luminance (6.3 cd/m²; Weber contrast = 0.05), participants did not make enough goal-directed saccades towards targets at 10° (without desensitisation P1/P2: *μ* = 217/193 ms, *σ* = 49/0 ms, *N* = 4/1 out of 40) and 20° (without desensitisation P1/P2: *μ* = 339/410 ms, *σ* = 84/96 ms, *N* = 7/7 out of 40), making it impossible to calculate the reaction-time differences in these conditions. At 6.6 cd/m² (Weber contrast = 0.10) and 10° eccentricity, the luminance used in experiments 1 and 3, reaction-time differences in means (Fig. 5A) reached up to ~100 ms and variability (Fig. 5D) of ~30 ms. These differences diminished rapidly with increasing luminance and were negligible beyond 7.3 cd/m² (Weber contrast = 0.22).

In contrast, for the most central target at 5° (Fig. 5B), only the lowest target luminance (6.3 cd/m^2^; Weber contrast = 0.05) resulted in increases in reaction-time mean (P1: 36 ms, CI = [24,47] ms, MoE = 12 ms, *p* < 0.001; P2: 42 ms, CI = [29,55] ms, MoE = 13 ms, *p* < 0.001 for both participants) and negligible increases in variability (Fig. 5E). For the peripheral targets at 20° (Fig. 5C, F), the measured differences were lower than 20 ms for all luminance values. Crucially, the desensitising effect was most pronounced under low-luminance conditions, where visual processing is inherently more challenging and became negligible at intensities of 7.3 cd/m² (Weber contrast = 0.22) and above.

To evaluate how stimulus intensity modulates the desensitisation effect, an LMEM was fitted with target luminance as a primary factor (Table 2). Under baseline conditions, representing targets at 10° eccentricity with a luminance of 6.6 cd/m² (Weber contrast = 0.1) and no desensitisation, the predicted reaction time was 214 ms.

**Table 2:** Summary of fixed-effects and the interaction effect from the linear mixed-effects model for Experiment 2 predicting promptness (1/s).

Fixed effects	*β* (1/RT, ms)	SE	95% CI (1/RT)	*p*
(Intercept)	4.68, 214	0.115	[4.45, 4.90]	<0.001
Desensitisation	–0.46, 23	0.030	[–0.52, –0.40]	<0.001
Target Eccentricity (ref = 10°)				
5°	0.01, –1	0.028	[–0.05, 0.06]	0.83
20°	–0.46, 23	0.029	[–0.51, –0.40]	<0.001
Desensitisation × Target Eccentricity (ref = No Desensitisation × 10°)				
Desensitisation × 5°	0.37, –16	0.041	[0.29, 0.45]	<0.001
Desensitisation × 20°	0.23, –10	0.042	[0.15, 0.31]	<0.001
Target Luminance (ref = 6.6 cd/m^2^)				
6.3 cd/m^2^ (lowest luminance)	–1.01, 58	0.033	[–1.08, –0.95]	<0.001
13.2 cd/m^2^ (highest luminance)	0.83, –33	0.029	[0.78, 0.89]	<0.001

The intercept represents the baseline performance at the reference levels of each variable (no desensitisation, 10° target eccentricity and 6.6 cd/m^2^ target luminance), with all other coefficients representing additive deviations from this state. Significant effects (*α* = 0.01) highlight the spatial and luminance parameters that most robustly influenced saccadic reaction times (RT).

In line with Experiment 1, the largest reaction time delays occurred within the 10° desensitised area as confirmed by the interaction between desensitisation and eccentricity. In contrast, desensitisation had a significantly reduced impact on reaction times for targets located outside this region at 5° (–16 ms) and 20° (–10 ms).

Stimulus intensity exerted a strong influence on oculomotor performance, with reaction times decreasing consistently as target luminance increased. Responses ranged from ~272 ms for the dimmest targets (6.3 cd/m²; Weber contrast = 0.10) to ~181 ms for the brightest targets (13.2 cd/m²; Weber contrast = 1.20).

These findings underscore that light-induced desensitisation selectively impairs visual responsiveness, particularly for low-contrast stimuli within the targeted retinal zone. Given that the dimmest targets often failed to elicit goal-directed saccades, a luminance of 6.6 cd/m² (Weber contrast = 0.1) was identified as the optimal stimulus for subsequent experiments, as it provided the most sensitive measure of desensitisation-induced delays.

#### Experiment 3—Spatial Extent of Desensitisation

While the previous two experiments examined the effect of desensitisation solely along the horizontal dimension, clinical visual-field tests are usually performed in two-dimensional space. Therefore, localised desensitisation was investigated across the visual field in both radial and angular dimensions. Specifically, targets were presented in eight directions across three eccentricities, either with or without desensitisation. A 2-s desensitisation duration was used with a 150-ms target delay, 6.6 cd/m² target luminance (Weber contrast = 0.10) and a 200-ms pre-desensitisation period.

#### Two-Dimensional Map of Reaction Times

**Fig. 6:**
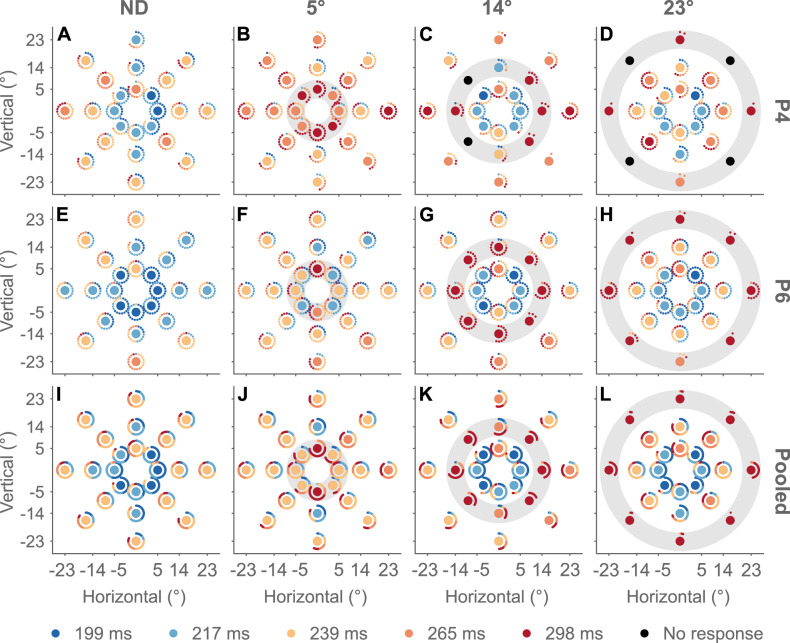
Reaction time map for all desensitisation conditions (ND, 5°, 14° and 23°) for two participants (P4 and P6; **A**–**H**) and all participants pooled (‘Pooled’; **I**–**L**). The desensitisation ring is indicated as a grey circular patch. Large, coloured circles depict mean reaction times, derived from the corresponding promptness distributions. Smaller circles indicate reaction times from individual repetitions. The extent to which the smaller circles surround the larger circle reflects the percentage of goal-directed saccadic responses across all repetitions of a target location. That is, 3 and 6 o’clock indicates that 25% and 50% of target presentations elicited goal-directed saccades, respectively. Colours represent reaction time bins based on the 10th, 30th, 50th, 70th and 90th percentiles of the pooled participants’ promptness distribution. Black circles indicate that the target failed to elicit a goal-directed saccade. ND = no desensitisation.

Figure 6 provides an overview of the reaction times without (‘ND’) and with desensitisation (5°, 14° and 23°) across the visual field for two example participants (P4 and P6; Fig. 6A–H) and pooled across all participants (Fig. 6I–L). Mean reaction time per location, derived from the corresponding promptness distributions, is indicated by the large, coloured circles. Cold and warm colours indicate faster and slower reaction times, respectively. Per location, reaction times of individual repetitions are indicated, with smaller circles surrounding the larger circle. In line with experiments 1 and 2, without desensitisation (ND; Fig. 6A, E, I) reaction times were shortest (200–220 ms) for central locations and tended to increase towards the periphery (240–270 ms). Desensitisation increased reaction times up to ~300 ms within desensitised regions and could decrease the proportion of goal-directed saccades, especially at an eccentricity of 23° for the two example participants, but also in the pooled data (Fig. 6D, H, L). Overall, all participants showed a response pattern in line with the hypothesis that saccadic reaction times within desensitised areas are increased.

#### Effect of Desensitisation on Detection Rate

**Fig. 7:**
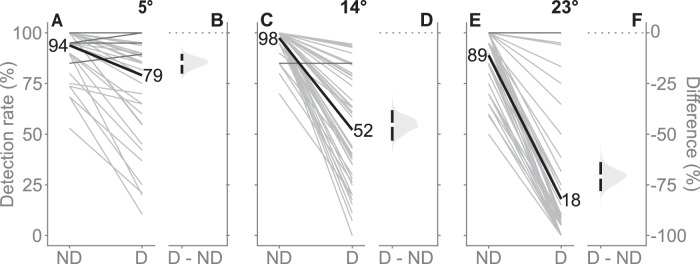
Effect of desensitisation on detection rate (i.e., the percentage of goal-directed saccades). Thin lines in panels **A**, **C** and **E** show the difference in detection rate between trials with (‘D’) and without (‘ND’) desensitisation for individual participants and directions, with light grey lines representing decreases and dark grey lines indicating increases. Group means are shown by thick black lines. Panels **B**, **D** and **F** display the corresponding mean-difference (‘D–ND’) plots, with white markers representing differences and black bars 95% confidence intervals. D = desensitisation; ND = no desensitisation.

Estimation statistics were used to quantify the impact of desensitisation at the three tested eccentricities on the detection rate, i.e., percentage of goal-directed saccades across all 20 repetitions. For each eccentricity, the detection rate was compared between conditions with desensitisation (‘D’) and without desensitisation (‘ND’). Each pair in the line plots of Fig. 7A, C and E represents the comparison for a single participant and target. The estimation statistics for differences (‘D–ND’) in mean detection rate and corresponding bootstrap distributions are depicted in mean-difference plots (Fig. 7B, D and F).

At 5° eccentricity (Fig. 7A and B), desensitisation led to a modest reduction in detection rate, with a mean decrease of 15% (95% CI = [10,20]%, MoE = 5%, *p* < 0.001). At 14° (Fig. 7C and D), this effect was more pronounced, with the detection rate dropping to 45% (95% CI = [38,53]%, MoE = 8%, *p* < 0.001). The most pronounced reduction was observed at 23° eccentricity (Fig. 7E and F), where the detection rate decreased to 71% (95% CI = [64,78]%, MoE = 7%, *p* < 0.001). These results demonstrate that desensitisation substantially impairs the ability to generate saccades toward the target, and that this impairment becomes more severe with increasing target eccentricity.

To evaluate how desensitisation affected target detection across the visual field, the detection rate was modelled as a function of both target and desensitisation eccentricity (Table 3). Under baseline conditions (targets at 5° without desensitisation), detection rates were high, with goal-directed saccades occurring in ~94% of repetitions.

**Table 3:** Summary of fixed-effects and the interaction effect from the linear mixed-effects model for Experiment 3 predicting detection rate, i.e., percentage of goal-directed saccades across all 20 repetitions.

Fixed effects	*β* (%)	SE	95% CI (%)	*p*
(Intercept)	93.87	5.383	[83.30, 104.44]	<0.001
Target eccentricity (ref = 5°)				
14°	3.63	3.371	[–2.99, 10.25]	0.28
23°	–4.96	3.371	[–11.58, 1.66]	0.14
Desensitisation eccentricity (ref = No Desensitisation)				
5°	–14.80	3.371	[–21.42, –8.17]	<0.001
14°	–12.53	3.371	[–19.15, –5.91]	<0.001
23°	–9.80	3.371	[–16.42, –3.18]	0.003
Target eccentricity × desensitisation eccentricity (ref = 5° × no desensitisation)				
14° × 14°	–32.83	4.767	[–42.19, –23.46]	<0.001
23° × 23°	–61.00	4.767	[–70.41, –51.68]	<0.001
14° × 5°	10.57	4.767	[1.20, 19.93]	0.03
14° × 23°	2.29	4.767	[–7.07, 11.65]	0.63
23° × 5°	3.44	4.767	[–5.93, 12.80]	0.47
23° × 14°	–9.90	4.767	[–19.27, –0.54]	0.04

The intercept represents the baseline performance at the reference levels (5° target eccentricity and no desensitisation), with all other coefficients representing additive deviations from this state. Significant effects (*α* = 0.01) highlight the spatial parameters that most robustly influenced detection rate.

While desensitisation significantly reduced the likelihood of a successful saccade across all tested locations, its impact was highly dependent on the spatial relationship between the target and the desensitised zone. The impairment was most severe when the target and desensitisation were collocated. Specifically, detection rates decreased by 15%, 45% and 71% for targets presented at 5°, 14° and 23° eccentricity, respectively. In contrast, when desensitisation was applied at non-collocated eccentricities (last three rows of Table 3), the impact on detection was substantially smaller or negligible, further highlighting the spatial specificity of the functional deficit.

#### Effect of Desensitisation on Reaction Time and Variability

**Fig. 8:**
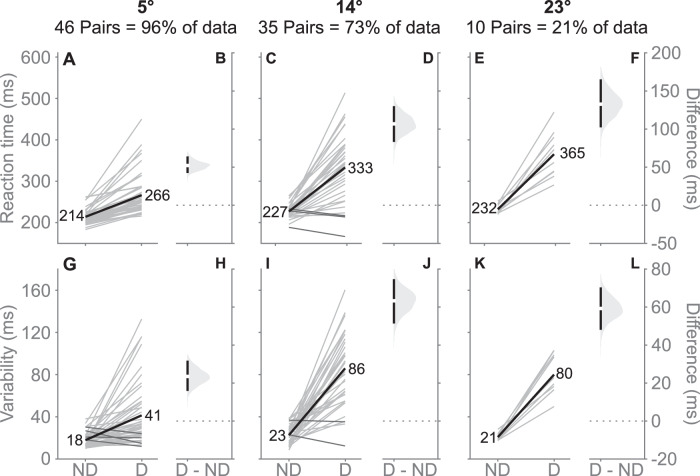
Desensitisation increases both reaction time (**A**–**F**) and reaction time standard deviation (variability, **G**–**L**) across eccentricities 5°, 14° and 23°. Panels **A**, **C**, **E**, **G**, **I** and **K** show comparisons for individual participants and directions between no-desensitisation (‘ND’) and desensitisation (‘D’) conditions. Light and dark grey lines depict increases and decreases after desensitisation, respectively. The black line indicates the group mean. The number of included data pairs is indicated above each plot, along with the percentage of available data retained after excluding undirected saccades. Panels **B**, **D**, **F**, **H**, **J** and **L** display the difference (‘D–ND’) for each parameter (white notch) with bootstrap-derived 95% confidence intervals (black bars within grey distribution).

Figure 8 shows the effect of desensitisation on mean reaction time and its variability across the three tested eccentricities, using estimation statistics in the same format as Fig. 7. At all eccentricities, desensitisation caused a marked increase in both the mean and standard deviation of reaction time. Mean reaction time (Fig. 8A–F) was slower in the desensitisation condition compared with the no-desensitisation condition. At 5°, reaction time increased by 52 ms (95% CI = [42,64] ms, MoE = 11 ms, *p* < 0.001), at 14° by 107 ms (95% CI = [83, 130] ms, MoE = 24 ms, *p* < 0.001) and at 23° by 133 ms (95% CI = [102, 165] ms, MoE = 32 ms, *p* = 0.003). A similar pattern was observed for the standard deviation of reaction times (Fig. 8G–L), indicating greater variability under desensitisation.

To evaluate how the spatial overlap between the target and desensitised region influenced reaction times, an LMEM was fitted with target and desensitisation eccentricity as primary factors (Table 4). Under baseline conditions, representing targets at 5° with no desensitisation, the predicted reaction time was ~213 ms. Responses slowed as target eccentricity increased, with reaction times increasing by ~19 ms at 14° and ~23 ms at 23°, relative to the 5° baseline.

**Table 4:** Summary of fixed-effects and the interaction effect from the linear mixed-effects model for Experiment 4 predicting promptness (1/s).

Fixed effects	*β* (1/s, ms)	SE	95% CI (1/s)	*p*
(Intercept)	4.70, 213	0.072	[4.56, 4.84]	<0.001
Target eccentricity (ref = 5°)				
14°	–0.38, 19	0.027	[–0.43, –0.33]	<0.001
23°	–0.45, 23	0.027	[–0.50, –0.39]	<0.001
Desensitisation eccentricity (ref = no desensitisation)				
5°	–0.83, 46	0.029	[–0.88, –0.77]	<0.001
14°	–0.07, 3	0.028	[–0.13, –0.01]	0.01
23°	–0.09, 4	0.028	[–0.15, –0.04]	0.001
Target eccentricity × desensitisation eccentricity (ref = 5° × no desensitisation)				
14° × 14°	–1.16, 70	0.045	[–1.24, –1.07]	<0.001
23° × 23°	–1.40, 90	0.060	[–1.52, –1.29]	<0.001
14° × 5°	0.72, –28	0.040	[0.64, 0.79]	<0.001
14° × 23°	–0.13, 6	0.039	[–0.20, –0.05]	0.001
23° × 5°	0.70, –28	0.041	[0.62, 0.78]	<0.001
23° × 14°	–0.16, 7	0.041	[–0.24, –0.08]	<0.001

The intercept represents the baseline performance at the reference levels (ref = 5° target eccentricity and no desensitisation), with all other coefficients representing additive deviations from this state. Significant effects (*α* = 0.01) highlight the spatial parameters that most robustly influenced saccadic reaction times.

Retinal desensitisation significantly increased reaction times, with the magnitude of the impairment critically dependent on the spatial overlap between the target and the desensitised region. When targets were collocated within the desensitised zone, the delays were substantial and grew with eccentricity: ~46 ms at 5°, ~111 ms at 14° and ~149 ms at 23° compared to baseline. In contrast, for non-collocated targets, the impairment was significantly smaller, ranging from only ~3 to ~36 ms, demonstrating that the functional deficit was localised with only weak spill-over effects.

In summary, desensitisation significantly affected both the detection rate and mean reaction time and its variability in a spatially specific manner. These results are consistent with a physiologically grounded, local impairment, where the strongest functional deficits are confined to the desensitised area.

## Discussion

A reversible method was developed and validated for modelling the behavioural effects of glaucoma in healthy individuals. By desensitising localised retinal regions using non-harmful light patterns presented on a standard TFT monitor, functional impairments were induced that mirror the saccadic reaction-time characteristics seen in the disease. Saccadic reaction time is a known marker of functional impairment in glaucoma [7–12]. Across three oculomotor experiments, saccadic reaction times and detection rates were quantified under varying temporal, luminance and spatial conditions, with and without desensitisation. The findings demonstrated that localised desensitisation robustly increased both the mean (50–130 ms) and variability (20–60 ms) of saccadic reaction times, with effects decaying rapidly within 1 s and exhibiting strong spatial specificity, i.e., the largest impairments occurred when targets were presented at desensitised eccentricities. Detection rates at desensitised eccentricities could be as low as 0%, confirming that desensitisation induced localised functional deficits resembling scotomas observed in glaucoma.

The present approach provides a non-invasive, temporally reversible method to mimic the localised processing delays characteristic of glaucoma in healthy participants. It can facilitate rapid prototyping of reaction-time-based diagnostic tools such as EMP by enabling early-stage testing in healthy participants, who are more readily available than patients. Importantly, it reduces the burden on clinical populations by avoiding unnecessary testing during the initial development of new protocols. Additionally, it offers a controlled and reversible way to simulate visual-field deficits, supporting the design of paradigms aimed at early detection and modelling of conditions such as glaucoma.

### Temporal, Luminance and Spatial Dynamics of Retinal Desensitisation

Experiment 1 established that brief, localised light exposure induces reliable and reversible saccadic reaction-time delays that decay within 1 s. A 2-s exposure to the desensitising light is sufficient to produce robust behavioural effects. This efficiency minimises total testing time and participant fatigue, enhancing the method’s scalability and viability for clinical and research protocols.

Experiment 2 established that desensitisation-induced delays are luminance-dependent, primarily affecting dim targets. Since these effects become negligible above 7.3 cd/m² (0.22 Weber contrast), the method selectively disrupts low-contrast processing while preserving high-contrast responsiveness. This interaction models behavioural consequences of glaucoma, as performance deficits become more pronounced when patients are required to detect low-contrast rather than high-contrast stimuli [40].

Experiment 3 established that light-induced desensitisation produces localised functional impairments. When the target was presented within the desensitised zone (collocated), substantial reaction-time delays of 111 ms at 14° eccentricity and 149 ms at 23° eccentricity were observed. In contrast, when targets appeared outside the desensitised region (non-collocated), the increases in reaction time were significantly smaller, ranging between only 3 and 36 ms. This four-fold difference in effect size demonstrates that the functional impairment is concentrated within the targeted retinal region. The presence of only minor ‘spill-over’ effects suggests that while the desensitisation is highly localised, it may engage adaptive gain-control mechanisms that have subtle, secondary influences on adjacent retinotopic areas [41, 42]. The spatial precision of these effects indicates they are driven by a topographically specific, physiologically grounded adaptation process, likely photopigment bleaching [19] or retinal adaptation [43], rather than global factors such as attention or fatigue [44]. Because this spatial selectivity mirrors the localised nature of glaucomatous visual-field loss [45], the method serves as a reproducible behavioural proxy for modelling clinical deficits and for optimising diagnostic paradigms that require precise functional mapping.

### Physiological Mechanisms Underlying Retinal Desensitisation Effects

Observed saccadic reaction-time effects are consistent with retinal adaptation, specifically photopigment bleaching [19, 23, 25] and gain control [41, 42]. These results align with classical findings where intense light exposure reduces photoreceptor sensitivity by depleting visual pigment [19, 23–25]. Although classical studies used high-intensity Ganzfeld sources (~5 × 10⁷ cd/m²), a 2-s exposure from a 350 cd/m² TFT screen induced detectable delays followed by rapid recovery [21, 24]. Since the cone system saturates at ~200 cd/m² and recovers in minutes [46, 47], unlike the rod system, which requires ≥30 min [48], the present findings likely reflect cone-system engagement, including pigment regeneration and phototransduction reactivation [49].

Desensitisation was spatially specific, as saccadic reaction time and detection rate impairments were confined to desensitised regions. This selectivity supports retinotopically organised adaptation over global attentional or motivational factors [44]. Beyond photoreceptor bleaching, these deficits are likely modulated by adaptive gain-control in the retina and early visual cortex [43, 50], which optimises coding but transiently suppresses sensitivity in recently stimulated areas. Increased reaction-time variability may reflect local gain-control fluctuations or partial recovery during the response window.

Following desensitisation, participants reported brief afterimages matching the desensitisation area’s shape, consistent with retinal photonegative aftereffects caused by localised photopigment depletion [51–53]. Though their exact mechanisms remain debated [54–59], these afterimages likely reflect retinal adaptation potentially modulated by cortical processes. Their persistence for tens of seconds, contrasting with the 1–2 s decay of saccadic reaction-time effects, suggests that functional impairments are more transient than full photopigment recovery. Since inter-trial intervals (median: 11.8–21.2 s) exceeded recovery times significantly and afterimages did not interfere with non-desensitised targets, they functioned as perceptual byproducts rather than confounds to the reaction-time data.

Peripheral regions exhibited stronger desensitisation, aligning with retinal heterogeneity. Declining cone and photopigment densities [60, 61] contribute to regional adaptation differences, with peripheral cones adapting more strongly and rapidly than central ones [62]. Increased photoreceptor signal convergence and larger receptive fields beyond ~15° eccentricity [63–65] likely drive this heightened peripheral susceptibility. Such structural and functional heterogeneity is essential to consider when designing and interpreting visual-field manipulation paradigms.

Taken together, the saccadic reaction-time effects observed across all experiments are in line with a transient, localised reduction in retinal sensitivity due to mild photopigment bleaching, likely amplified by downstream gain-control processes. These mechanisms operate over short time scales and with high spatial precision, making them ideally suited for use in modelling early-stage visual-field deficits such as those seen in glaucoma.

### Methodical Considerations

This light-based desensitisation provides a distinct alternative to established simulation paradigms. Unlike gaze-contingent masking [66], which offers spatial precision but requires low-latency hardware, vignetting or static overlays [67], which lack gaze alignment, the current method is retina-fixed. This avoids the technical complexity and screen-space artefacts inherent in digital masks or immersive virtual or augmented reality setups [68]. By utilising a photobleaching mechanism directly on the retina, a more ecologically consistent and accessible model of the localised saccadic reaction-time increases is provided.

Unlike digital gaze-contingent masks that can produce visible artefacts in complex naturalistic scenes, the present photobleaching method preserves the integrity of the external image by inducing the functional deficit internally at the photoreceptor level. This ensures the ‘scotoma’ is perceived as a natural loss of information rather than a digital overlay, significantly enhancing ecological validity. Consequently, this approach is uniquely suited for studying functional deficits during real-world tasks, such as reading or scene navigation, where maintaining the visual environment’s integrity is essential.

The eye tracker used here sampled at 60 Hz, which is suboptimal for detailed saccade kinematics and may bias peak velocity, duration and to a lesser extent saccadic latencies [69–71]. The use of Akima interpolation and velocity‑threshold detection improved the stability of onset estimation, but cannot overcome the intrinsic temporal resolution of 16.7 ms. Thus, absolute saccadic reaction time values should be interpreted with this limitation in mind. However, because sampling‑related distortions apply equally across conditions, it is expected that the main conclusions, which are based on relative saccadic reaction time differences between conditions, will remain valid.

A ring-shaped desensitisation area was employed to mitigate target prediction. A localised patch would have provided a spatial cue, potentially confounding reaction times through focused expectation [31, 44]. The desensitisation area preserved the requirement for stimulus-driven saccades by allowing targets to appear at randomised meridians. This configuration also facilitated internal validation. Targets at non-desensitised eccentricities (5° and 20°) served as controls, confirming that impairments were spatially specific rather than a result of global fatigue or inattention [44]. While optimal for validation, rings lack the clinical realism of irregular glaucomatous scotomas, such as arcuate or paracentral defects [3, 45]. Transitioning to complex scotoma morphologies is a necessary next step to enhance EMP prototyping for mapping the intricate spatial patterns characteristic of the disease.

### Comparison to Visual-Field Loss Typical of Glaucoma

The desensitisation method presented here offers a practical approach for modelling the behavioural consequences of localised visual deficits in healthy observers. While it does not reproduce the neurodegenerative pathophysiology of glaucoma, it captures the saccadic reaction-time characteristics in a controlled and reversible manner. Specifically, the method induces increases in both reaction time mean and variability, similar to those observed in glaucoma [7–13]. The observed 50–130-ms increases in the mean are well within the range reported for glaucoma-like visual-field loss, with typical delays between 40 and 200 ms when assessed with EMP and related oculomotor paradigms [12, 13]. Importantly, the desensitisation method reproduces not only the slowing but also the increased reaction-time variability characteristic of glaucomatous processing [13]. This variability has been interpreted as a behavioural correlate of reduced signal-to-noise ratio in degraded visual channels [13]. The present data support this interpretation. Together, these parallels suggest that controlled retinal desensitisation provides a reversible model of behaviours associated with visual-field deficits that can accelerate the optimisation of EMP paradigms and other reaction-time-based diagnostic tools.

However, the current method operates at the level of photoreceptors, inducing transient and spatially confined desensitisation via photopigment bleaching and gain control mechanisms. Glaucoma is a progressive neurodegenerative disease primarily affecting retinal ganglion cells (RGCs), leading to irreversible damage to the optic nerve and permanent, localised visual-field loss [3, 72]. The deficits induced by this method are not due to structural damage but rather to a temporary reduction in photoreceptor sensitivity, allowing for full recovery within seconds. This reversibility is a central strength of the method, enabling repeated within-subject measurements and controlled manipulation of deficit location and severity without long-term consequences.

Another key distinction is related to brain plasticity. In glaucoma, the brain undergoes functional adaptation to gradually altered input, potentially including cross-modal plasticity, where non-visual modalities recruit deafferented visual cortex regions [73–75]. In contrast, the short duration and transient nature of the desensitisation protocol preclude such long-term cortical reorganisation. This isolation from broader neural plasticity ensures that behavioural changes observed during the task reflect immediate sensory limitations, not compensatory processes that evolve over time.

An additional difference lies in the specificity of impairment. Damage associated with glaucoma is largely indiscriminate with respect to visual features such as colour, contrast or spatial frequency, as it results from the degeneration of RGCs with broad functional roles [76, 77]. In contrast, the current method could, in principle, be refined to selectively desensitise specific photoreceptor subtypes, for example, through chromatic adaptation targeting cones with different spectral sensitivities [23, 78]. This targetability opens new avenues for experimental designs that probe feature-specific processing under localised visual impairment.

Lastly, this method can be applied to desensitise the two eyes asymmetrically in a stereoscopic setting [13]. This would allow for the systematic study of binocular summation [79] under acute ocular asymmetry, potentially not only providing insight into the impact of glaucoma on binocular processing [80, 81], but also for the testing of binocular models [82].

## Conclusion

In summary, while the present transient retinal desensitisation method does not replicate the exact cellular origin or chronic progression of glaucoma, it offers a safe, flexible and well-controlled means of modelling the spatially localised behavioural consequences of glaucoma. Specifically, desensitisation induces a significant increase in mean reaction time (50–130 ms) and reaction-time variability (20–60 ms), alongside a marked decrease in goal-directed saccades. In extreme cases, particularly at greater eccentricities, these impairments result in localised detection deficits resembling functional scotomas. The method’s primary utility lies in accelerating the development of visual-field assessment tools by providing a predictable behavioural proxy for visual deficits. Its physiological basis in photopigment bleaching, combined with its reversibility and spatial specificity, makes it particularly well suited for rapid prototyping of visual-field assessment tools and for studying the early-stage behavioural impact of localised visual deficits.

## Data Availability

The dataset and analysis code are available in the figshare repository: https://figshare.com/s/69672bdf8e31175486dd.
